# The hot pick-up technique for batch assembly of van der Waals heterostructures

**DOI:** 10.1038/ncomms11894

**Published:** 2016-06-16

**Authors:** Filippo Pizzocchero, Lene Gammelgaard, Bjarke S. Jessen, José M. Caridad, Lei Wang, James Hone, Peter Bøggild, Timothy J. Booth

**Affiliations:** 1 Center for Nanostructured Graphene (CNG), DTU Nanotech, Technical University of Denmark, DK-2800, Kongens Lyngby, Denmark; 2 Kavli Institute (KIC) at Cornell for Nanoscale Science, Cornell University, Ithaca, New York 14853, USA; 3 Department of Mechanical Engineering, Columbia University, New York, New York 10027, USA

## Abstract

The assembly of individual two-dimensional materials into van der Waals heterostructures enables the construction of layered three-dimensional materials with desirable electronic and optical properties. A core problem in the fabrication of these structures is the formation of clean interfaces between the individual two-dimensional materials which would affect device performance. We present here a technique for the rapid batch fabrication of van der Waals heterostructures, demonstrated by the controlled production of 22 mono-, bi- and trilayer graphene stacks encapsulated in hexagonal boron nitride with close to 100% yield. For the monolayer devices, we found semiclassical mean-free paths up to 0.9 μm, with the narrowest samples showing clear indications of the transport being affected by boundary scattering. The presented method readily lends itself to fabrication of van der Waals heterostructures in both ambient and controlled atmospheres, while the ability to assemble pre-patterned layers paves the way for complex three-dimensional architectures.

## Abstract (web summary)

The reliable assembly of two-dimensional materials into van der Waals heterostructures is a critical step towards nanoscale electronic integration. Here the authors present a technique for batch fabrication of graphene/boron nitride stacks with clean interfaces and high-yield.

## Graphical abstract

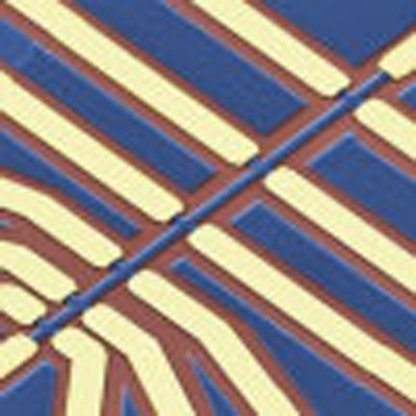

The controlled isolation and assembly of single- and few-layer sheets of two-dimensional (2D) materials into van der Waals (vdW) heterostructures has thrown open the doors for the design and fabrication of new devices and functionalities based on 2D materials with an unprecedented flexibility and atomic precision. The perspectives are astounding, with applications ranging from electronics^1^,^2^, photovoltaics^3^,^4^ and sensing^5^,^6^ through to memory storage^7^ with the intriguing possibility of increased performance and a wide range of functionalities on flexible, transparent substrates^8^,^9^.

Clean interfaces between 2D materials result in the best device performance^10^,^11^,^12^,^13^,^14^,^15^ – any contaminants present between interfaces usually gather up as ‘blisters' between layers, leading to deterioration of transport properties^16^ as well as compromising the perfect vdWs interlayer adhesion between the layers. Devices can be produced in controlled atmospheres such as in gloveboxes^17^ but without special cleaning steps to remove ad-layers and careful monitoring, contamination and decreased interlayer adhesion may still be an issue. Oxygen plasma pre-treatment increases the number and area of individual flakes of 2D materials produced by micromechanical exfoliation on oxidized silicon substrates^18^, but results in increased substrate interaction, which dopes graphene and is manifested as a reduced Raman *I*(2D)*/I*(*G*) peak ratio^19^.

We present here room temperature mobility measurements from a batch-fabricated set of 22 mono-, bi and trilayer blister-free encapsulated graphene field effect devices – assembled in ambient atmosphere–with over 280 individual metal-graphene contacts. We find that blisters of trapped interfacial contamination commonly observed in such samples by optical and atomic force microscopy can be completely eliminated by stacking individual 2D crystals into vdWs heterostructures at temperatures of 110 °C, even in ambient atmosphere. We prove that the reduction of the *I*(2D)*/I*(*G*) ratio in graphene due to plasma treatment of the cleavage substrate is fully reversed when the crystals are lifted from this substrate, meaning that plasma pre-treatment is a viable way to obtain large and pristine 2D material flakes for integration into vdWs heterostructures. By actively tuning the interfacial adhesion and cleanliness through temperature whilst completely avoiding any contact with liquids in the stacking procedure, we are able to controllably pick up and drop-down 2D materials, including single-layer crystals that have been pre-patterned using electron-beam lithography (EBL). This method enables us to produce a statistically significant data set of field effect mobility measurements from 22 mono-, bi- and trilayer encapsulated graphene devices with >280 contacts. Seven of the 16 monolayer devices and 55% of the measurements display carrier mean-free paths comparable or exceeding the channel width, with carrier mean-free paths limited by boundary scattering.^20^ Bi- and trilayer devices show diffusive behaviour with average mobilities above 20,000 and 15,000 cm^2^ V^−1^ s^−1^, respectively. No annealing at high temperatures is necessary to obtain this high performance^13^,^21^,^22^.

## Results

### Exfoliation of 2D materials and Raman precharacterisation

**Figure 1:**
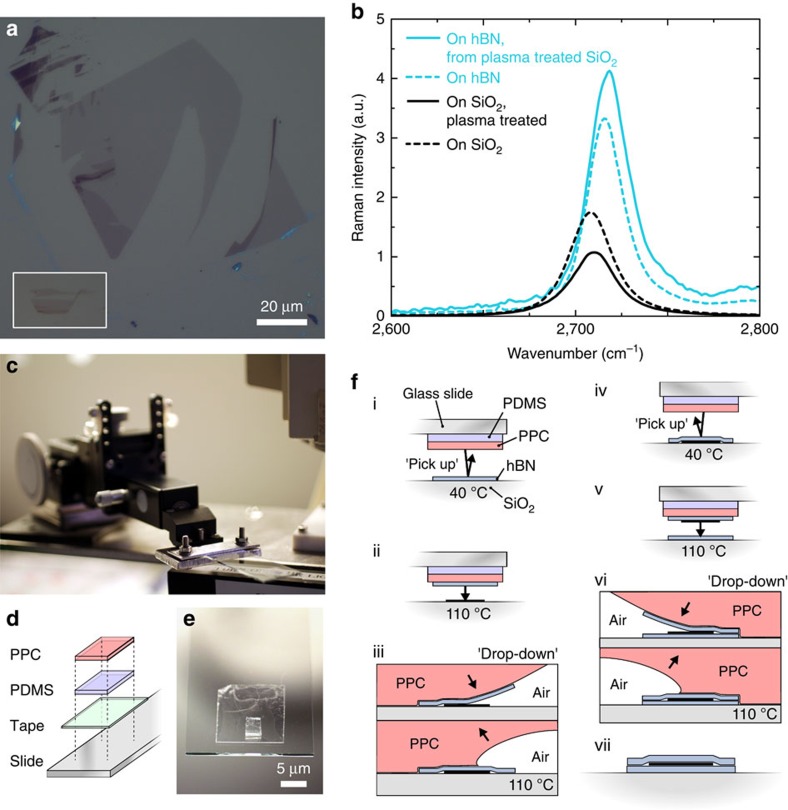
Assembly and precharacterization of heterostructures. (**a**) Monolayer graphene flakes produced on oxygen plasma-treated SiO_2_. Inset (white square): typical graphene flake size on non-plasma-treated SiO_2_. To scale with (**a**). (**b**) Graphene Raman 2D peak dependence on substrate – the Raman *I*(2D)*/I*(*G*) ratio increases for graphene picked up with hBN from SiO_2_ irrespective of the oxygen plasma pre-treatment of the substrate. (**c**) Micromanipulator with slide assembly used for assembly of heterostructures. (**d**,**e**) Polymer stack on glass slide used for pick-up and drop-down. (**f**) Schematic process flow for assembly of 2D heterostructures by pick-up and drop-down.

Mono-, bi- and trilayer graphene and thin (thickness in the range 20±10 nm) hexagonal boron nitride (hBN) flakes, all with areas of over 2,000 μm^2^, are produced on oxidized silicon substrates by micromechanical cleavage. A combination of oxygen plasma pre-treatment of the substrate and thermal release of the adhesive tape^18^ is employed to increase the size and number of the crystals produced - exfoliation on non-plasma-treated substrates yields smaller and fewer crystals (Fig. 1a). Plasma treatment is known to introduce trapped charges in oxidized silicon, while heating increases substrate conformity and induces roughness in graphene^23^. Both of these effects can potentially impact device performance. The Raman *I*(2D)*/I*(*G*) peak ratios of graphene flakes cleaved on plasma-treated oxide show a noticeably higher doping than for graphene on untreated oxide (*I*(2D)*/I*(*G*) peak ratio=1.1 on plasma-treated versus 1.8 on untreated–Fig. 1b). This trend is not present in the same graphene flakes when lifted from the substrate using hBN crystals. For such samples, the *I*(2D)*/I*(*G*) peak ratio increases from <2 to 4.2 and 3.3 for graphene from plasma-treated and untreated SiO_2_, respectively – Fig. 1b. This shows that plasma cleaning of the substrate can be used to produce more and larger 2D crystals, and has no permanent impact on those crystals after lifting them from the production substrate.

### Heterostructure assembly

**Figure 2:**
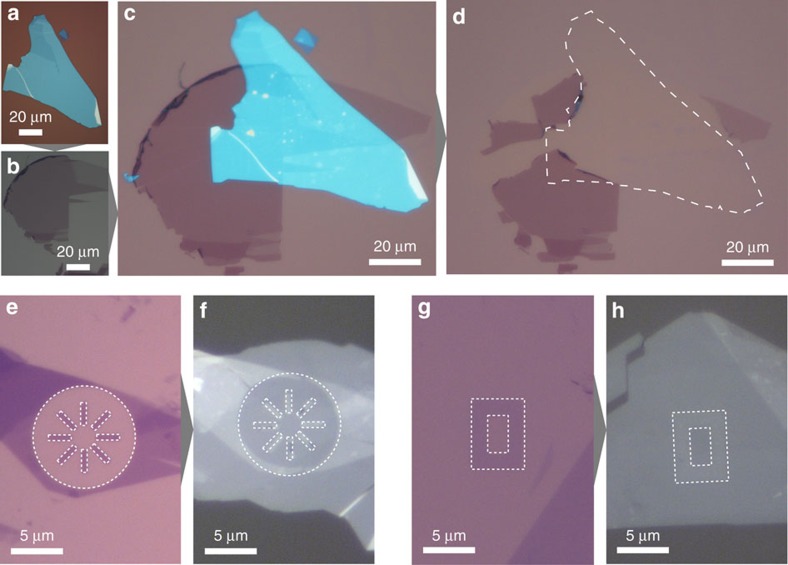
Adhesion between pristine and patterned 2D materials. (**a**) hBN flakes are dropped down onto (**b**) graphene flakes (single and bilayer shown) to produce (**c**) a stack. (**d**) Interlayer adhesion between the hBN and graphene is sufficient to selectively tear the graphene away from the substrate. The dashed line indicates the previous extent of the hBN flake before pick-up. (**e**) A graphene monolayer which has been pre-patterned into eight rectangles (dark areas inside dashed lines) with EBL. (**f**) The graphene strips have been picked up by a hBN crystal and dropped down. The graphene is now the bright areas inside the dashed lines. Panels **g**,**h** same as **e**,**f** with a rectangular graphene frame. This approach allows multiple stacking of laterally patterned two-dimensional layers into complex three-dimensional heterostructures.

We use a polypropylene carbonate (PPC) coated polydimethylsiloxane (PDMS) block mounted on a glass slide to capture (pick-up) and release (drop-down) 2D materials (Fig. 1d,e). For full details of the described techniques, see Supplementary Methods. The slide and polymer block is positioned in *x*, *y* and *z* using a micromanipulator (Fig. 1c) fixed to a heated microscope stage. By tuning the temperature above the boiling point of water and the glass transition temperature of the polymer, flakes can be reproducibly picked up or dropped down at desired positions (Figs 1f, 2a–c). Higher temperatures during transfer are enabled by the use of oxygen plasma treatment of the PDMS before application of the PPC layer, which increases the adhesion between the PPC and the PDMS, and prevents delamination at high temperatures.

Temperatures above 110 °C favour the vdWs adhesion between hBN and graphene, whereas a temperature of 40 °C is sufficient to lift hBN from oxidized silicon cleavage substrates using the PPC/PDMS block. We note that assembly at high temperatures produces adhesion forces between graphene and hBN which are strong enough to tear the graphene flake, leaving behind graphene regions which are not covered by hBN adhering to the oxidized silicon substrate (Fig. 2a–d). Such high-adhesion forces lead to a pick-up yield of close to 100%. It is always possible to pick up hBN from SiO_2_ using PPC, regardless of the temperature, but we are unable to pick up graphene using PPC in the same way – graphene adheres more strongly to the oxidized silicon surface than the PPC, leading to the tearing of edges that can be seen in Fig. 2d. Such structures are a result of the reported preferred tearing directions in monocrystalline graphene^24^. A temperature of 110 °C is required to pick up graphene from plasma-treated oxidized silicon using an hBN crystal on PPC – at 40 °C the graphene adhesion to the plasma-treated SiO_2_ surface dominates. For graphene on SiO_2_ surfaces that have not been plasma treated, hBN can be used to pick up the graphene flakes regardless of temperature, as previously reported^10^. Lithographically pre-patterned 2D materials can also be reliably picked up for subsequent encapsulation in this way (Fig. 1e–h), which allows a greater flexibility in device architecture.

A key feature of this technique is the ability to produce large numbers of encapsulated graphene samples in parallel, by covering many graphene flakes on their production substrate with dropped down hBN flakes (Fig. 1f (i–iii)), subsequently picking these hBN/graphene stacks up, and finally dropping them down on new hBN flakes produced on oxidized silicon substrates (Fig. 1f (iv–vii)). This enables the rapid batch fabrication of many heterostructure stacks such as those shown here, and the parallelization of subsequent processing steps of lithography and etching to define devices.

### Avoidance of blisters during assembly

We compare the number and size of blisters formed during stacking at 110 versus 40 °C using optical and atomic force microscopy, and find that the cleanliness of the interface critically depends on both the speed of the approach of the flakes and the temperature. One point of the PPC/PDMS block touches the target substrate first, due to the slide being tilted by a few degrees in the micromanipulator. Full contact between these two surfaces is made controllably in all cases, limiting the speed of the contact area front to <1 μm s^−1^ by controlling the *z*-height of the stage.

**Figure 3:**
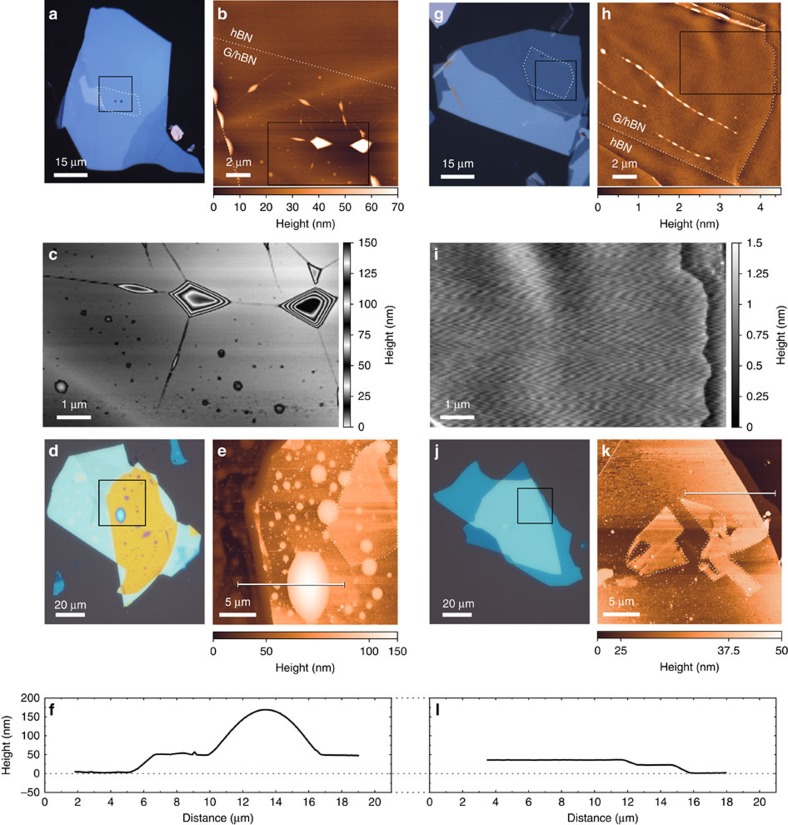
Atomic force microscopy characterization of blister formation at different assembly temperatures. (**a**) Optical image of graphene/hBN stack on PPC (after step iv in Fig. 1f), picked up from pristine SiO_2_ at 40 °C. (**b**) AFM topography image of region indicated in (**a**) – blisters are visible across the stacked region in AFM with heights of ≈50 nm. (**c**) The marked region in **b** with periodic colourscale to emphasize height variations across blisters. (**d**) Optical image of G/hBN stack dropped down onto a second hBN flake after approaching at 40 °C on SiO_2_ (step vii in Fig. 1f). Many hemispherical blisters are visible over the stack surface. (**e**) AFM topography image. Encapsulated graphene flake is visible, indicated by dotted line. (**f**) topography line profile of blister from indicated region in **d**. Panel **g** as **a**, but with pick-up performed at 110 °C. (**h**) AFM image shows smaller overall height variation and large blister-free regions. (**i**) The marked region in **h** have two orders of magnitude less height variation compared with the region **c** of same size. Panel **j** as **d**, but with drop-down performed at 110 °C. No trapped blisters are visible optically or (**k**) in the AFM scanned region. Encapsulated few-layer graphene regions are indicated. (**l**) AFM line profile from **i** showing uniform height across the stack.

Stacking at 40 °C results in apparently blister-free heterostructures, but on heating above 70 °C these blisters become visible and mobile, increase in size, agglomerate and then stabilize within a few seconds (Supplementary Movies 1, 2 and 3). These blisters are also afterwards apparent between the graphene and hBN (Fig. 3a–c), and in the finished fully encapsulated graphene sample (Fig. 3d–f). The blisters are up to 100 nm tall and 10 μm across, and evenly distributed over the sample, and account for an area fraction of 20% or more of the total stack area (Fig. 3e). In contrast, assembling at the higher temperature of 110 °C results in a complete absence of such blisters (Fig. 3g–l), leading to vdWs heterostructure devices with greatly reduced interfacial contamination. The surface height variation of the stack is much reduced (Fig. 3i), and no blisters of contamination are visible optically or by atomic force microscope (AFM) (Fig. 3h,i,k,l). We note that the encapsulated graphene flakes are visible in Fig. 3e,k–any trapped interfacial contamination in the stack must therefore either consist of a continuous and homogeneous layer, or be entirely absent.

**Figure 4:**
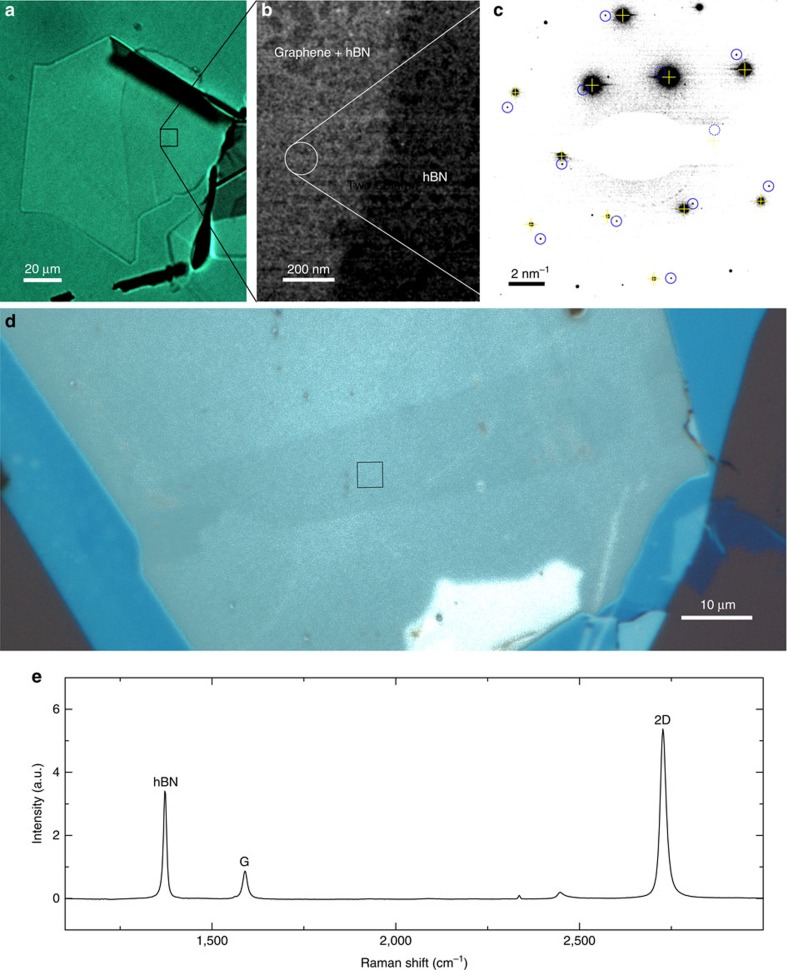
TEM and Raman spectroscopy. (**a**) Optical transmission image of a graphene flake adhered to hBN on PPC/PDMS. (**b**) Dark-field tilted beam TEM image of region indicated in **a**. A discontinuous layer of amorphous carbon is visible. The objective aperture has been positioned to provide lighter contrast from the graphene region. (**c**) Selected area electron diffraction pattern from region indicated in **b**. The contrast has been enhanced to show both the monolayer graphene diffraction pattern (reflections indicated with blue circles) alongside the hBN diffraction pattern (indicated with yellow crosshairs). Points masked by the beam stop or by nearby stronger reflections are indicated with dashed versions of the same. (**d**) Enhanced contrast optical image of the encapsulated graphene. The boxed region indicates the region where the spectrum in **e** was acquired. Maps of the *I*(2D)*/I*(*G*) ratio and G peak full width at half maximum (FWHM) are available in Supplementary Fig. 6. (**e**) Raman spectrum of hBN-encapsulated graphene fabricated by the hot pick-up method, normalized to the G peak intensity. The *I*(2D)*/I*(*G*) peak ratio is 5.8 (with Lorentzian fits to the peaks (*R*^2^>0.994) indicating a ratio of 6.12±0.02), with a FWHM of 14.2±0.5 cm^−1^.

In attempt to detect the presence of trapped contamination within stacks, we transferred graphene adhered to hBN to silicon nitride transmission electron microscopy (TEM) aperture grids using the same drop-down methods described above. An optical image of the hBN-graphene heterostructure adhered to PPC/PDMS is shown in Fig. 4a. We then performed tilted beam dark-field TEM imaging of the samples by selecting one of the first-order graphene reflections. This causes the graphene and any materials with a similar lattice spacing to appear bright in the image. Figure 4b shows enhanced contrast of graphene with respect to hBN, in addition to an incomplete layer of amorphous carbon which is usually present on 2D materials^25^,^26^, and commonly observed in TEM experiments. While we are unable to strictly exclude the possibility that this amorphous carbon is trapped between the layers, we note that it is present everywhere in the dark-field image in Fig. 4b, and has not segregated into blisters (which would cause enhanced dark-field contrast) as has been observed previously in vdWs heterostructures. Figure 4c shows a selected area diffraction pattern of the region indicated in Fig. 4b, where both hBN and monolayer graphene reflections are visible. The lattices have a relative rotation of ∼7.5° for this sample, showing that the hBN and graphene lattices have not displayed the self-rotation or ‘snap-in' behaviour recently reported in hBN-encapsulated graphene heterostructures.^21^

Raman data for a representative hBN-encapsulated graphene sample in Fig. 4d is shown in Fig. 4e, with an enhanced contrast optical image of the heterostructure inset showing the collection region. We find a consistent *I*(2D)*/I*(*G*) ratio of >5, approaching 6, as previously shown for hBN-encapsulated graphene heterostructures, and a *G* peak full width at half maximum of 14±0.5 cm^−1^ over 5–10 μm areas of the heterostructures. Raman maps showing the spatial variation of these values are provided in the Supplementary Information.

### Carrier mobility measurements

**Figure 5:**
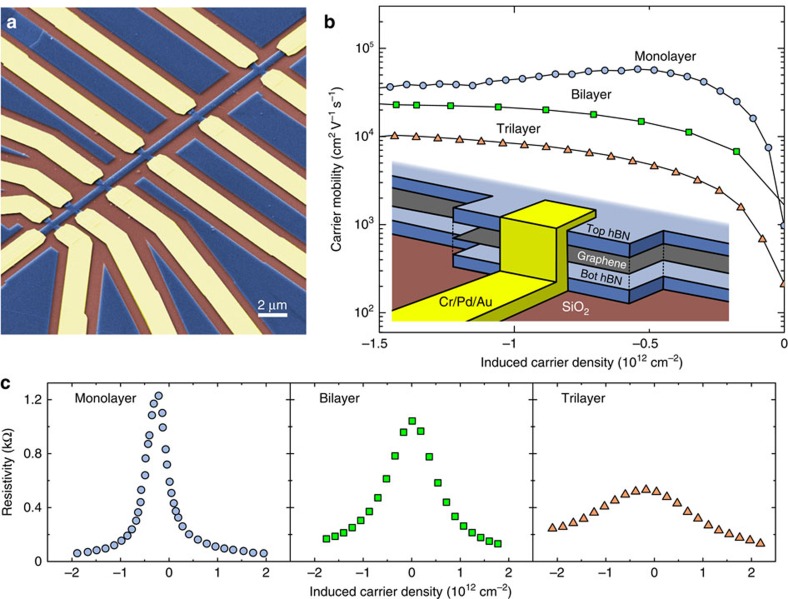
Electrical measurements of Hall bar devices. (**a**) False colour scanning electron micrograph of encapsulated graphene device. Blue regions are unetched hBN and hBN-encapsulated graphene, brown are exposed SiO_2_, and yellow regions are metal contacts. (**b**) Room temperature field effect mobility measurements for representative monolayer (#4), bilayer (#11) and trilayer (#14) Hall bar devices. A monolayer device displaying diffusive transport is shown. Blue, green and orange indicate monolayer, bilayer and trilayer results, respectively. Inset shows exploded schematic of contact region. (**c**) Resistivity versus gate-induced carrier density for monolayer, bilayer and trilayer graphene Hall bar devices. Colour scheme as in **b**.

We batch fabricate hBN-encapsulated graphene field effect devices with edge contacts in both Hall bar and van der Pauw geometries from blister-free vdWs heterostructures stacks via EBL, plasma etching and metal deposition (Fig. 5a). SF_6_ plasma is used to etch, which we find shows significantly improved selectivity for hBN relative to Poly(methyl methacrylate) (PMMA; >45:1), graphene (>90:1) and SiO_2_ (>90:1), rather than the more commonly used CHF_3_ etch^10^,^11^.

Gated pairwise direct current two-point measurements of 284 contacts gives 251 working connections with contact resistances of a few kΩ·μm at the charge neutrality point, decreasing to a few hundreds of Ω μm away from the charge neutrality point (Supplementary Fig. 3), with residual carrier densities <10^12^ cm^−2^ at zero gate voltage for all monolayer, bilayer and trilayer graphene devices (Fig. 5b,c and Supplementary Fig. 4). All our measurements are performed at room temperature with a constant source-drain bias of 5 mV. The total yield of contacts – over 88% – is to our knowledge the largest reported to date in vdWs heterostructures devices considering the number of contacts produced.

**Figure 6:**
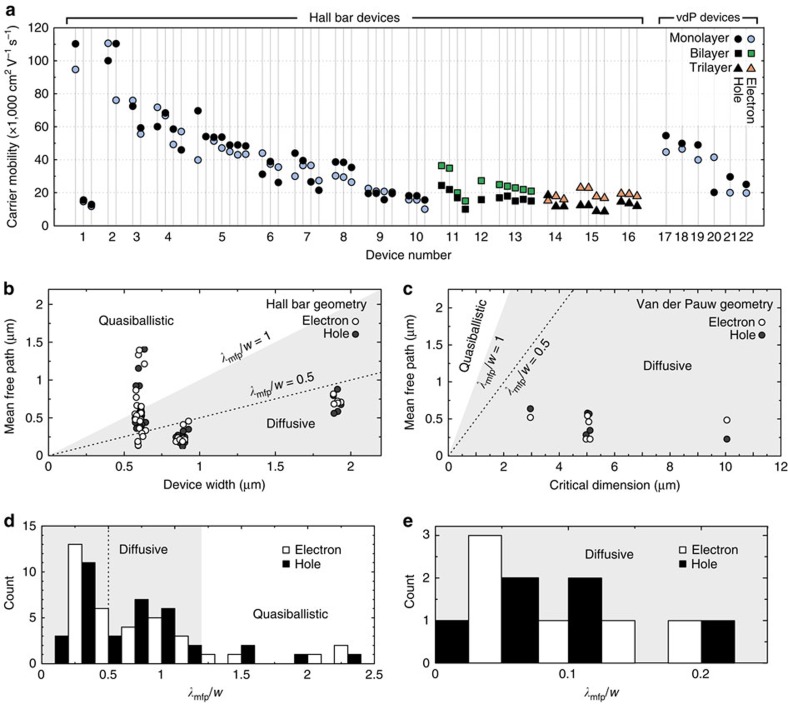
Mobility and mean-free path statistics. (**a**) Room temperature field effect carrier mobility plot for all the devices (Hall bar and van der Pauw) in this study. (**b**) Plot of electron and hole mobility for all monolayer Hall bar devices. Quasiballistic (*λ*_mfp_≥*w*) and diffusive (*w*>*λ*_mfp_) regimes are indicated by the white and grey regions, respectively. Note that 60% of our narrow channel devices have a calculated mean-free path larger than *w*/2 (dashed line), a clear indication of transport affected by boundary scattering^17^. (**c**) Plot of electron and hole mean free path for all monolayer van der Pauw devices. Quasiballistic (*λ*_mfp_≥*w*) and diffusive (*w*>*λ*_mfp_) regimes are indicated by the grey region. The average mean-free path is 450±170 and 420±140 nm for holes and electrons, respectively. (**d**) Histogram of monolayer electron and hole mean free path for all Hall bar devices. (**e**) Histogram of monolayer electron and hole mean free path for all van der Pauw devices.

As a result, we are able to present a statistical ensemble of electrical measurements for encapsulated graphene Hall bar devices. Figure 6a shows room temperature electron and hole mobilities extracted from 55 transconductance measurements of hBN-encapsulated mono-, bi- and trilayer graphene devices. The values of the mobility for bi- and trilayer samples are extracted at saturation, while at the local maximum (induced carrier density of ∼0.5 – 1 × 10^12^ cm^−2^) for single-layer devices (Fig. 5b). For single-layer devices, nominal mobilities of up to 117,000 cm^2^ V^−1^ s^−1^ were measured, with averages of 44,500±26,000 and 42,000±24,000 cm^2^ V^−1^ s^−1^ for holes and electrons, respectively. All of the field effect mobility values for any pair of monolayer device contacts devices are above 10,000 cm^2^ V^−1^ s^−1^, with more than 86% above 20,000 cm^2^ V^−1^ s^−1^.

In the diffusive limit the mean-free path *λ*_mfp_ can be found from the semiclassical conductivity^22^,^23^ *σ*=(2*e*^2^/*h*)*k*_F_ *λ*_mfp_, where *k*_F_=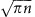 is the Fermi wave number, *n* is the carrier density, *e* is the elemental charge and *h* is Planck's constant. The high mobilities we calculate indicate that in the majority of cases in our monolayer devices the transport is limited by boundary scattering since the calculated mean-free path is comparable to or larger than *w*, the device width^20^ – in particular 70% of devices show *λ*_mfp_≥*w*/2 (Fig. 6b,d). Furthermore 16% of the measurements are strictly quasiballistic with the estimated mean-free paths exceeding the device width^27^,^28^.

Three bilayer and three trilayer graphene samples produced in the same way display field effect mobilities as high as 37,000 and 23,000 cm^2^ V^−1^ s^−1^, respectively. The mean field effect hole and electron mobilities are 17,000±4,000 and 24,500±7,000 cm^2^ V^−1^ s^−1^ for the bilayer devices, and 13,000±3,000 and 19,000±2,500 cm^2^ V^−1^ s^−1^ for the trilayer devices, respectively.

In addition to the Hall bar devices, we fabricated six encapsulated monolayer devices in van der Pauw (square) geometries of 3, 5 and 10 μm side length with four contacts placed at the corners of the device edges. For such devices we find hole and electron mobilities of 38,000±15,000 cm^2^ V^−1^ s^−1^ and 36,000±12,000 cm^2^ V^−1^ s^−1^ respectively. The average mean-free path for carriers in all such devices is 450±170 and 420±140 nm at room temperature. A representative gate sweep is available in the Supplementary Fig. 7 while the mean-free path of sample 2 is shown to reach 0.9 μm at a carrier density of 10^12^ cm^−2^ in Supplementary Fig. 8.

To prove the versatility of our technique, top-gated samples were fabricated by the drop-down of an additional hBN flake to cover the entire device area (Supplementary Fig. 5). This flake prevents short-circuiting during subsequent deposition of a metallic top-gate and contacts. The transconductance of the devices was compared (Supplementary Fig. 5) suggesting that top gates fabricated by drop-down are comparable in performance and quality to hBN and oxidized silicon bottom gates, which we attribute to the absence of trapped contamination.

## Discussion

The presented method for stacking 2D materials allows the preparation of clean heterostructures with a high throughput due to a higher yield of large exfoliated single-layer crystals, the parallelization of the different fabrication steps enabling batch production and with close to 100% pick-up yield.

The fact that the observed reduction of *I*(2D)*/I*(*G*) peak ratio in graphene flakes micromechanically cleaved on plasma-treated oxidized silicon is fully reversed on subsequent pick-up with hBN shows that oxygen plasma treatment of the substrate does not introduce point defects or charged contaminants attached to the exfoliated graphene. As a result, oxygen plasma pre-treatment is a suitable means of producing large numbers of appropriately sized exfoliated 2D materials on oxidized silicon for the fabrication of prototype vdWs heterostructures, which consistently exhibit clean interfaces and high carrier mobilities.

The complete elimination of blisters of contamination formed between flakes assembled at 110 °C versus the large number and size of blisters formed between flakes assembled at 40 °C or less, suggests that at low temperatures the surfaces of the flakes are covered by largely immobile adsorbants, which become more mobile at higher temperatures. This leads to the observed segregation of this layer into blisters during stacking at 40 °C and on subsequent heating above 70 °C. We find it extremely difficult to pick up crystals cleaved on plasma-treated substrates at 40 °C, despite an extended contact time of several hours, indicating that the adsorbants are effectively immobile in this case and interfere critically with the adhesion between flakes. The higher adsorbant mobility at 110 °C on the graphene surface allows them to diffuse across the surface of the 2D materials, away from the vdWs contact front which proceeds in a controlled way across the surface of the flakes during assembly, and appears to gather at the edges of flakes (Supplementary Movie 4)^22^. We infrequently observe inconsistent behaviour during drop-down in some samples (Supplementary Movie 5) that can lead to variation in the Raman *I*(2D)*/I*(*G*) ratio and *G* peak full width at half maximum of the encapsulated graphene in limited areas of the surface (Supplementary Fig. 6) – this highlights the importance of control of the drop-down step in making good contact between the layers of vdW heterostructures, here minimizing doping variations. Reduction in the viscosity of PPC at higher temperature may also allow a more conformal contact to be made between the 2D materials during stacking. It is interesting to note that the adhesion force between graphene and hBN resulting from stacking at temperatures above 110 °C is sufficient to overcome the adhesion of graphene to silicon oxide, but that adhesion between the PPC and the exposed graphene outside the hBN area is not strong enough to delaminate the graphene from the oxidized silicon. This points to the gradual exclusion of adsorbants or contaminants at the contact front between two 2D materials as the determining factor for optimization of adhesion forces and for establishing a good interlayer contact. This also raises the question of what materials the contaminants and adsorbants consist of. Since the process is performed in ambient atmosphere the three most likely sources of contamination are adsorbed water and/or trapped air, deposited ambient airborne hydrocarbon contamination^25^ or residues from the tape used for micromechanical cleavage produced during thermal release of the flakes on the substrate. The temperature of 110 °C needed to render these contaminants sufficiently mobile to be excluded from the advancing vdWs contact interface suggests adsorbed water as the most likely candidate. This would account for the observation of blister formation from apparently clean vdW heterostructures during heating to 70 °C – the adsorbed water coalesces as the vdW interface area increases, and vaporization of water causes the large volume blisters to be produced.

Atomic force microscopy shows that any residual contamination between layers in bubble-free regions must be either uniform or completely absent, since we are able to detect changes in thickness of the graphene encapsulated in hBN even down to a single monolayer (Fig. 3i,k). Dark field and selected area diffraction patterns from TEM studies (Fig. 4b,c) provide further evidence that the interface between the hBN and graphene does not include any contamination, along with providing evidence that the pick-up technique as described here does not result in self-aligning rotation of 2D materials^21^. Finally the Raman spectroscopy of graphene encapsulated in hBN (Fig. 4d,e), and Supplementary Fig. 6) shows a *I*(2D)*/I*(*G*) ratio approaching 6 and a *G* peak FWHM of 14 cm^−1^, which serve as evidence of a lack of doping of the graphene within the stack – results which are also confirmed by our electrical measurements of the low doping of our samples: all show residual carrier densities <10^12^ cm^−2^ at zero gate bias.

The presented techniques also enable us to pick up pre-patterned 2D materials, despite the presence of PMMA residues on top of the patterned flakes, an inevitable by-product of EBL. This means both that PMMA residues are largely excluded from the surface via this technique, and also that it is not a prerequisite of this technique to have atomically flat and clean surfaces before carrying out the stacking procedure, as presented previously^10^. This removes the limitation of simultaneous, through-stack lithography as the only option for the construction of vdWs heterostructures devices, allowing more complex and flexible architectures to be realised by enabling the clean stacking of separate, differently shaped 2D materials analogous to modern integrated circuits with two or more layers of active electronic components or interconnects in a single circuit^29^.

The devices produced using this hot stacking technique display very high room temperature mobilities, with 55% of the measurements in monolayer devices exhibiting mean-free paths larger than the half of the device width as well as uniformly low contact resistance. This high consistency is achieved without any annealing^10^,^12^,^16^,^21^ or further cleaning step of the stacks.

While little data is available in literature concerning the yield of heterostructure device fabrication, we believe that our stacking yield of nearly 100% and contact fabrication yield of 88% represent significant progress towards consistent, reproducible device fabrication of vdWs heterostructures. We find that statistically significant figures for these yields is unfeasible to obtain without batch fabrication, and that the high yield across many samples is a direct consequence of the complete exclusion of interfacial contamination during stacking at high temperatures. In comparison, stacking at lower temperatures lead to blisters occurring over an interface area fraction of 20% or more which strongly decreases the fabrication yield.

The mean carrier mobilities measured in bilayer and trilayer graphene devices of 21,000±7,000 and 16,000±4,000 cm^2^ V^−1^ s^−1^, with peak values of 37,000 and 23,000 cm^2^ V^−1^ s^−1^, are consistent with the highest values reported at room temperature in the literature for bilayers^30^ and are the highest reported for trilayers at room temperature^31^.

We note that in all but one instance, the electron mobility exceeds the hole mobility for bilayer and trilayer samples (Fig. 6a). This trend is largely reversed in monolayer samples, and is consistent with published observations^32^.

In 16% of the measurements the calculated room temperature mean-free path exceeds the sample width^10^,^20^,^33^ (Fig. 6b,d). In this limit, however, the semiclassical transport model used to estimate the mean-free path is no longer strictly valid, implying that both the highest values of mean-free path and the carrier mobility should be regarded with caution^20^. We speculate that the higher mobility and thus stronger tendency of ballistic transport behaviour for the monolayer samples is responsible for the larger spread in carrier mobility as compared with bi- and trilayers (Fig. 6a). The spread in carrier mobility is a consequence of transport in ballistic samples being more sensitive to boundary effects such as possible edge disorder and the exact device geometry^10^,^20^,^27^. The mean-free paths for the van der Pauw geometry samples (∼0.5 μm) are consistent with the onset of large variation in the calculated mobilities for the narrowest channel Hall bar samples (Fig. 6b–e), which supports the conclusion that boundary effects have a strong influence at such scales and lead to the observed large variations in the calculated mobilities here.

In conclusion, we have presented a facile and robust technique for the batch fabrication of vdWs heterostructures, demonstrated by the controlled production of 22 mono-, bi- and trilayer encapsulated graphene devices. Stacking at elevated temperatures (higher than 110 °C) results in high mean carrier mobilities for bilayer and trilayer samples of 21,000±7,000 and 16,000±4,000 cm^2^ V^−1^ s^−1^. The majority of monolayer Hall-bar devices exhibit transport limited by the edges, with mean-free paths exceeding half of the channel width. This stacking technique enables the pick-up and drop-down of flakes of 2D materials at desired locations with nearly 100% yield, and with a yield of 88% for subsequently fabricated electrical contacts with uniformly low contact resistance.

The absence of trapped contamination in our samples, manifesting as blisters between the stacked flakes, indicates that adsorbants are largely or completely excluded from between the flakes during the drop-down procedure, being pushed out in front of the proceeding vdWs contact region. In addition, the high temperature used for drop-down may also allow a more conformal contact between 2D materials during stacking as a result of reduction of viscosity of the PPC. The presented method readily lends itself to fabrication of any desired vdWs heterostructures, completely avoiding contact with liquids, whether in ambient conditions or in controlled atmospheres. The technique even permits the pick-up of lithographically patterned 2D materials and integration into vdWs heterostructures, or for additional layers to be dropped down onto pre-existing devices. By picking up device layers such as graphene with encapsulating layers such as hBN, the need for multiple separate encapsulation steps is avoided allowing efficient batch fabrication of heterostructure devices.

The ability to produce a large number of devices with high yield is a key advantage of this process, and paves the way for the statistical studies of device performance. Such studies are essential to gain an understanding of vdWs heterostructure based device performance in a technological perspective, for fundamental research and for further progress towards real-world device applications of 2D materials.

## Methods

### Cleaving of graphene and hBN

Graphene and hBN are cleaved on 100 nm Si oxide thermally grown on standard 4 inch Si wafers. Natural graphite crystals (NaturGrafit GmbH) and hBN bulk crystals (HQgraphene) were mechanically exfoliated with Nitto Denko SWT 20+ die sawing tape. Oxidized silicon is treated in oxygen plasma for 3 min (PlasmaEtch PE-50, 300 mbar O_2_, 120 W), and the 2D material loaded tape is immediately applied to the silicon oxide surface. The tape is subsequently released from the surface by heating to 85 °C on a hot plate.

### Preparation of the glass slide for pick-up and drop-down

The PDMS is prepared from SYLGARD 184 by mixing 10 parts base and 1 part curing agent and cured at 70 °C overnight, leading to a 1-mm-thick layer. The PDMS is treated with oxygen plasma as above for 10 min and then a PPC layer is spun on top of it (15% in anisole, 50 K, 1,500 r.p.m.), resulting in a thickness of 5 μm. Double-sided tape is used to attach a 1 × 1 mm^2^) piece of PPC coated PDMS to a glass microscope slide.

### Raman spectroscopy

Raman spectra are taken with a Thermo Fisher DXR Raman spectrometer using a 455-nm laser source with a power of 1 mW and 20 s duration multiple exposures. Raman spectra and maps for encapsulated graphene were acquired in a Thermo Fisher DXRxi Raman spectrometer using a 455-nm source, with a power of 10 mW and 20 s duration to provide adequate signal to noise ratio from the graphene within the heterostructure.

### Atomic force microscopy (AFM)

The AFM scans are done in a NTEGRA scanning probe microscope from NT-MDT with a Smena measuring head. The scans are performed in tapping mode with typical parameters of a driven frequency of 340 Hz, a magnitude of 10 nA, a set point of 5 nA and scan speed of 10–30 μm s^−1^.

### Transmission electron microscopy (TEM)

Tilted beam dark-field images were acquired in a Tecnai T20 G2 operated at 200 kV by selecting a first-order graphene reflection with an objective aperture with a diameter of 2 nm^−1^ in the diffraction plane. Selected area diffraction (SAD) patterns were taken in the same instrument with a 200-nm-diameter SAD aperture.

### Device fabrication

The EBL is performed in a JEOL JBX-9500FS, with an acceleration voltage of 100 kV. PMMA is used as resist in the EBL process, a 4-wt% PMMA 996K in anisole solution is spun on the chips (1 min at 1,500 r.p.m., acceleration 500 r.p.m. s^−1^) , followed by a post-bake (10 min at 150 °C).

### Etching

The etching of the stack is performed in a SPTS ICP Etch, O_2_ is used to etch the graphene and SF_6_ is used to etch the top and bottom hBN. Metal contacts of 2 nm Cr, 15 nm Pd and 30 nm Au are deposited by Physimeca ΦSES250 electron-beam evaporation with low rates (∼1 Å s^−1^ for Cr and Pd and ∼3 Å s^−1^ for Au).

### Data availability

The data that support the findings of this study are available from the corresponding author on request.

## Additional information

**How to cite this article:** Pizzocchero, F. *et al.* The hot pick-up technique for batch assembly of van der Waals heterostructures. *Nat. Commun.* 7:11894 doi: 10.1038/ncomms11894 (2016).

## Supplementary Material

Supplementary Information Supplementary Figures 1-14 and Supplementary Methods

Supplementary Movie 1 Trapping and migration of blisters in hBN/graphene/hBN. Mobile blisters within an hBNgraphene-hBN heterostructure on SiO_2_ assembled at 40°C and heated to 70°C.

Supplementary Movie 2 Trapping and migration of blisters after pick-up. Mobile blisters within an hBN-graphene heterostructure on PPC/PDMS before drop-down on hBN, assembled at 40°C and heated to 70°C.

Supplementary Movie 3 Trapping and migration of blisters after drop-down. Mobile blisters within an hBNgraphene heterostructure on SiO_2_ before pick-up assembled at 40°C and heated to 70°C.

Supplementary Movie 4 Blister-free drop-down. Drop-down of an hBN flake adhered to PPC/PDMS onto a graphene layer on SiO_2_. Control of the contact front forces any contamination between the faces of the 2D materials out of the heterostructure towards the edges.

Supplementary Movie 5 Blister formation during drop-down. Drop-down of an hBN flake adhered to PPC/PDMS onto a graphene layer on SiO_2_. In this instance, the contact front is not well controlled, resulting in the visible formation of blisters and inhomogeneous Raman *I(2D)/I(G)* and *G* peak FWHM values.
