# Supplementary material for: The hot pick-up technique for batch assembly of van der Waals heterostructures
Source: Nat Commun. 2016 Jun 16;7:11894. doi: 10.1038/ncomms11894 (PMC4912641; doi:10.1038/ncomms11894)
Supplement: Supplementary Information — Supplementary Figures 1-14 and Supplementary Methods [file ncomms11894-s1.pdf]

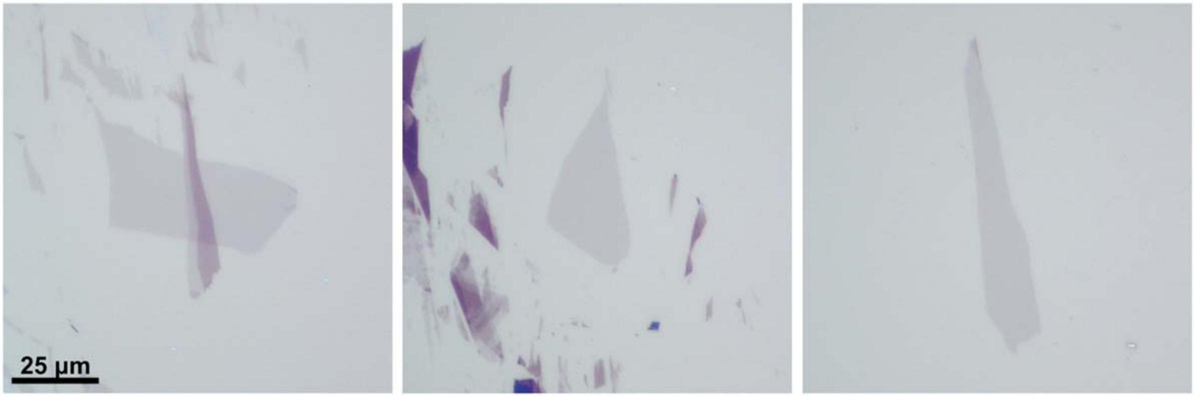

Supplementary Figure 1: Micromechanical cleavage of graphene on oxygen plasma treated Si/SiO_2. Optical microscopy images of three examples of large single layer graphene flakes cleaved on a single Si/SiO_2 chip after oxygen plasma treatment.

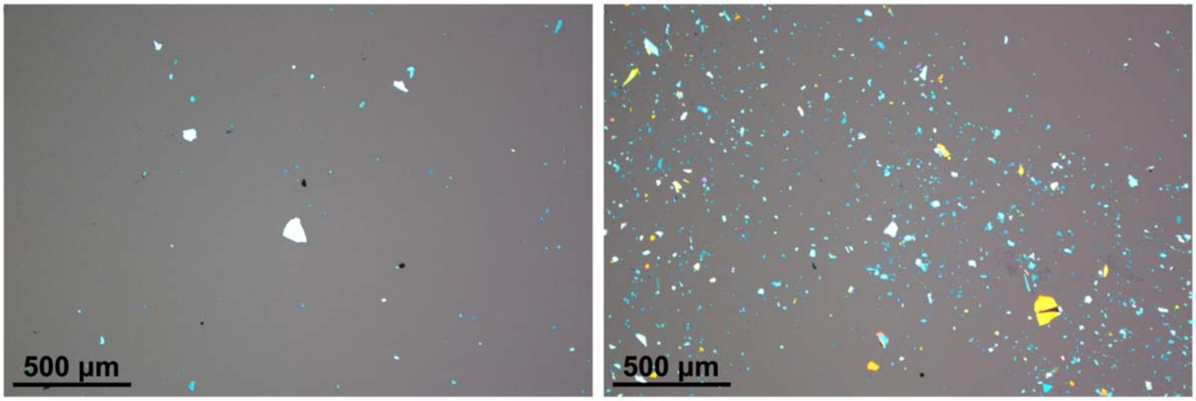

Supplementary Figure 2: Comparison of hBN yield. Optical images showing the difference in cleaving yield of hBN on pristine (left) and oxygen plasma-treated (right) SiO_2 surfaces, using the same cleaving tape.

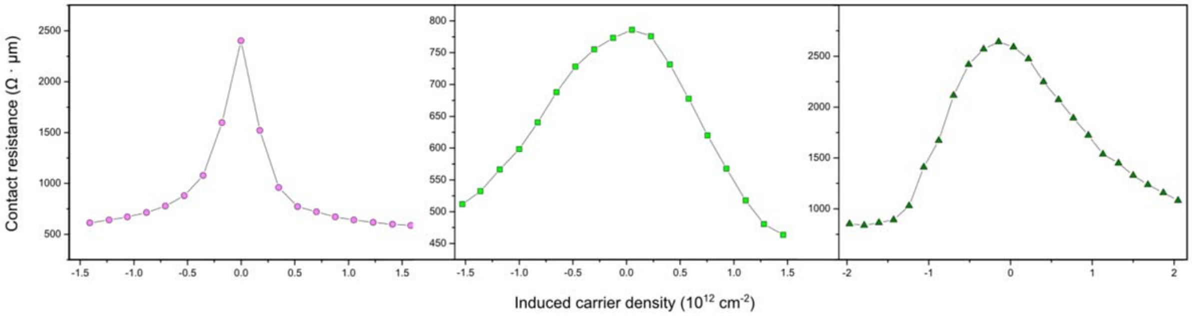

Supplementary Figure 3: Contact resistance measurements of encapsulated graphene. Contact resistance measurements from single- (left), bi- (middle) and trilayer (right) graphene devices. The values are extracted from the measurements of source-drain current and R_{xx}.

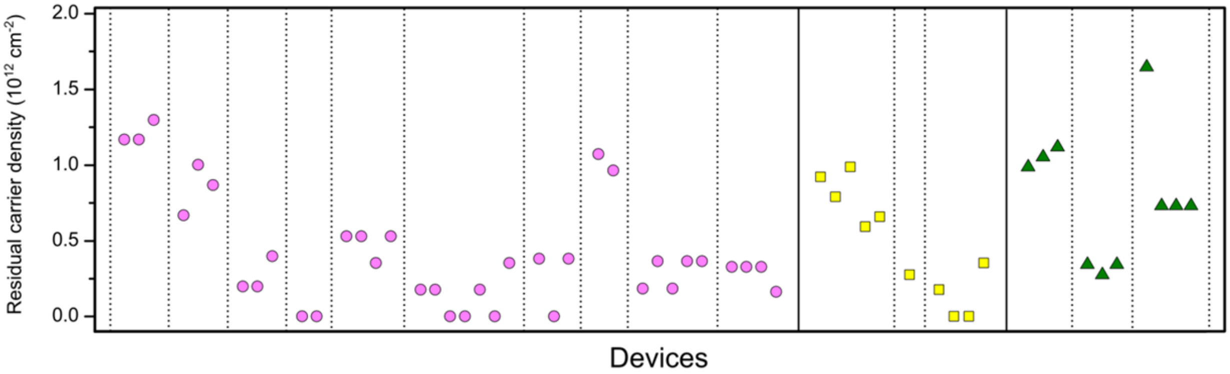

Supplementary Figure 4: Doping of encapsulated graphene. Residual carrier density at zero bias for the Hall bar device measurements reported in Figure 6.

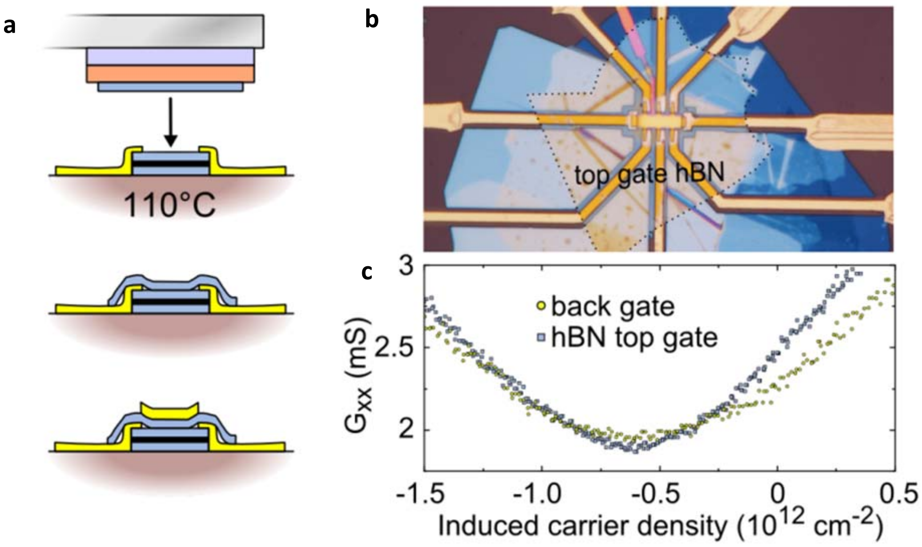

Supplementary Figure 5: Top gate addition. a) Schematic process flow for addition of hBN-insulated top gate. b) Optical image of top gate insulator hBN dropped down onto device after fabrication. c) Conductance vs. gate-induced carrier density for the same device using a Si back gate and a hBN top gate.

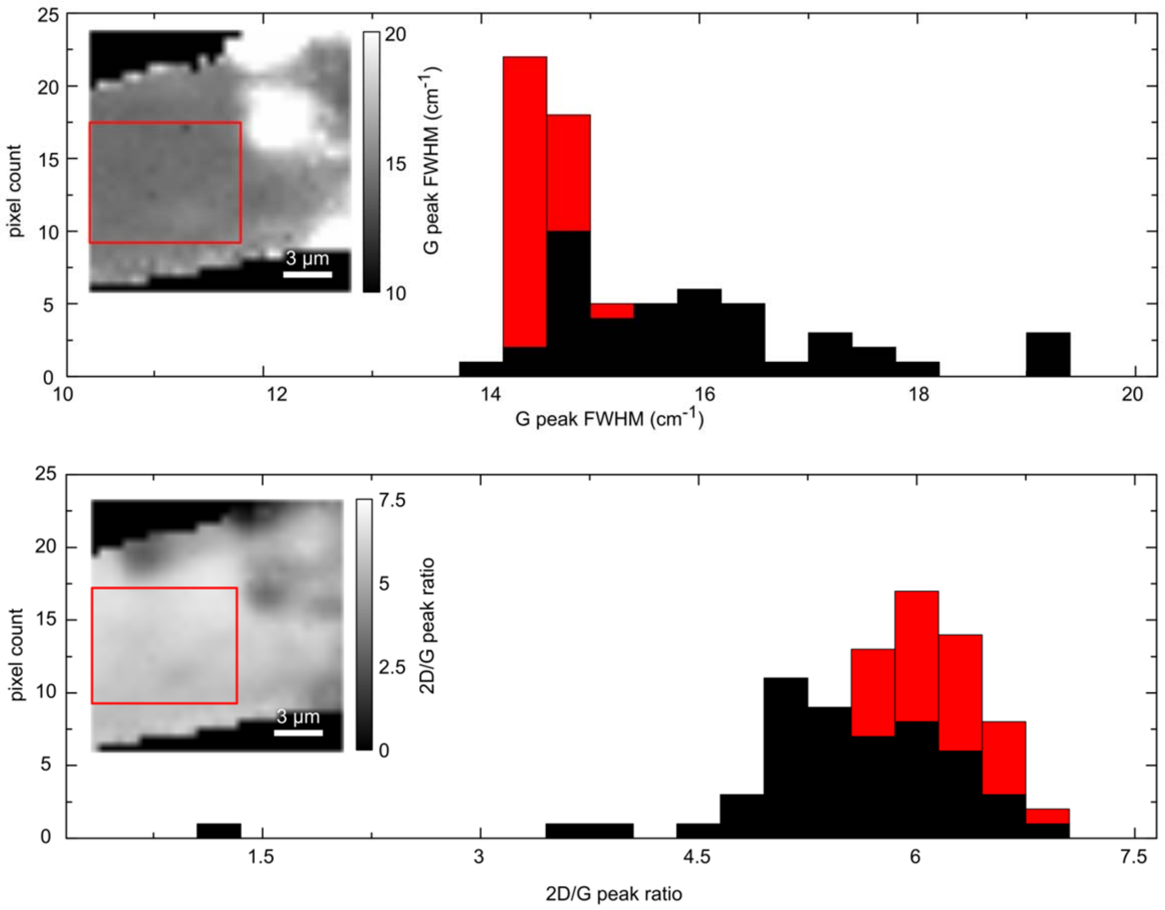

Supplementary Figure 6: Homogeneity of Raman characteristics. Raman maps of hBN encapsulated graphene, taken from the indicated region in Figure 4d inset in the main manuscript. Histograms show pixel counts of the measured parameters, the *G* peak full width at half maximum (FWHM) and *I(2D)/I(G)* peak intensity ratio. Contributions to the distributions from the boxed regions in the maps are marked in red in the histograms, whilst the black areas of the histograms represent the values outside of the areas marked in red. These regions are specifically chosen to show variation in the Raman properties of encapsulated graphene dependent on the control of the contact front (see Supplementary Movie 5). The drop down techniques used in this study result in large regions of encapsulated graphene with a narrow distribution of *G* peak FWHM around 14-15 cm^{-1} and an *I(2D)/I(G)* peak intensity ratio of around 6. These values demonstrate the low residual doping of such samples.

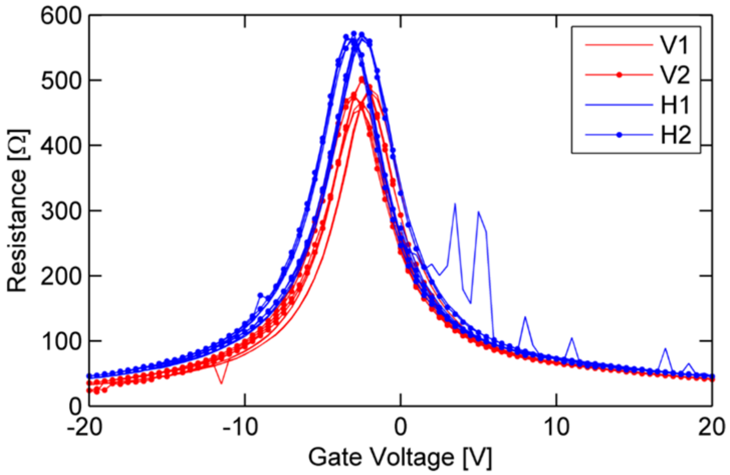

Supplementary Figure 7: van der Pauw measurements of square devices. Representative gate voltage sweep for a van der Pauw device with 5 µm side length – device #21 in Figure 6a of the main manuscript. Reproducible gate induced changes in the resistance irrespective of the configuration of the contacts (V1, V2, H1, H2) imply that the device is homogeneous and that the reciprocity theorem holds. See Supplementary Methods.

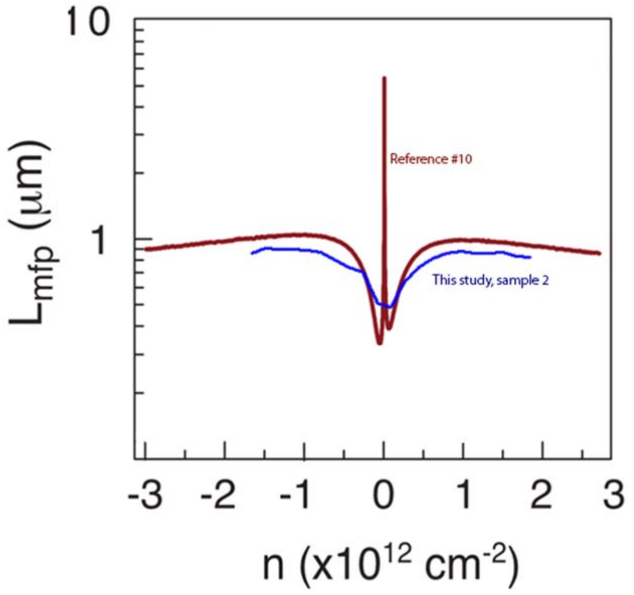

Supplementary Figure 8: Mean free path comparison. Comparison of mean free path in this study (blue curve) vs. data from reference 10 (main text references) (brown curve) from a 1.9 µm wide Hall bar device – device 2 in Figure 6a of the main manuscript. The figure is adapted from reference 10 (main text references).

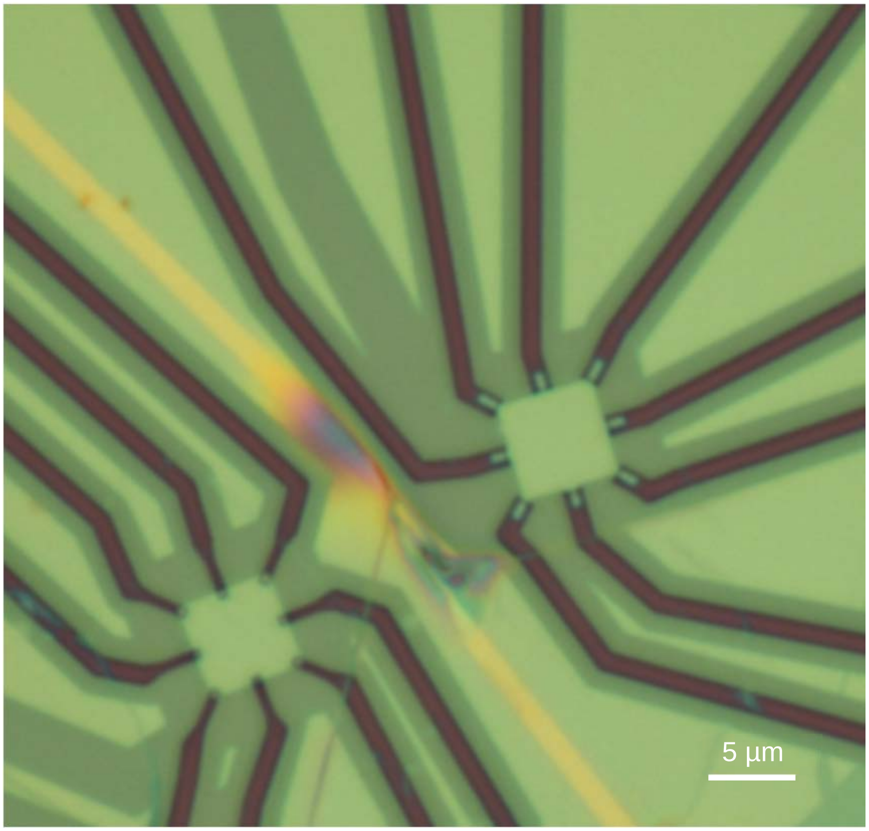

Supplementary Figure 9: Fabrication of van der Pauw devices. Optical image of two van der Pauw type encapsulated devices before deposition of metal electrodes and lift-off.

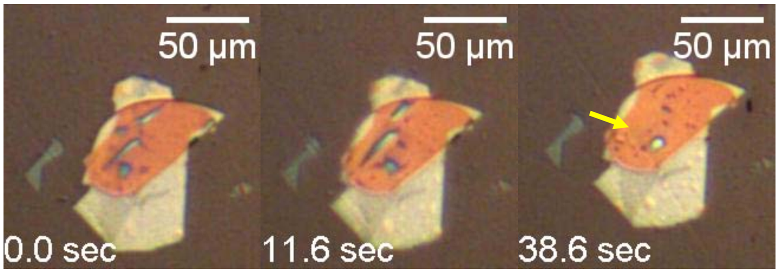

Supplementary Figure 10: Trapping and migration of blisters in hBN/graphene/hBN. Optical image of mobile blisters (indicated by yellow arrow) within an hBN-graphene-hBN heterostructure on SiO_2 assembled at 40°C and heated to 70°C (selected frames from Supplementary Movie S1).

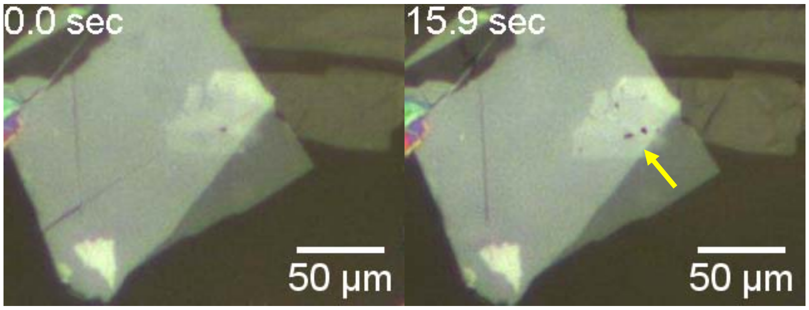

Supplementary Figure 11: Trapping and migration of blisters after pick-up. Optical image of mobile blisters (indicated by yellow arrow) within an hBN-graphene heterostructure on PPC/PDMS before drop-down on hBN, assembled at 40°C and heated to 70°C (selected frames from Supplementary Movie 2).

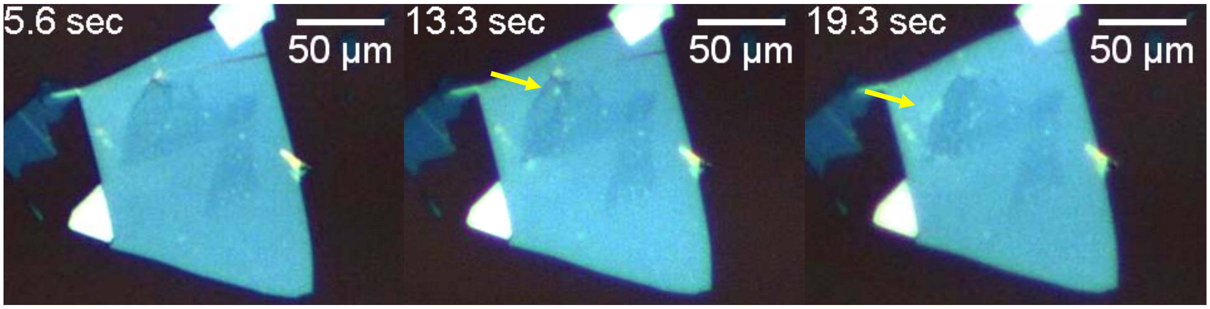

Supplementary Figure 12. Trapping and migration of blisters after drop-down. Optical image of mobile blisters (indicated by yellow arrow) within an hBN-graphene heterostructure on SiO_2 before pick-up assembled at 40°C and heated to 70°C (selected frames from Supplementary Movie 3).

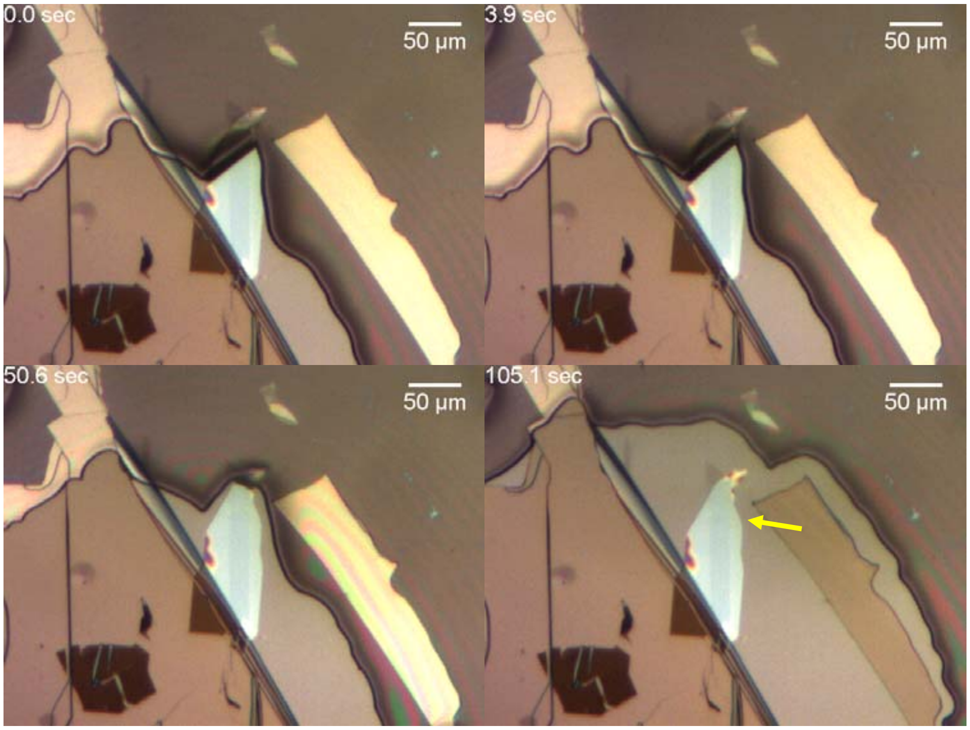

Supplementary Figure 13: Blister-free drop-down. Drop-down of an hBN flake adhered to PPC/PDMS onto a graphene layer on SiO_2. Control of the contact front forces any contamination between the faces of the 2D materials out of the heterostructure towards the edges (indicated by yellow arrow). Selected frames from Supplementary Movie 4.

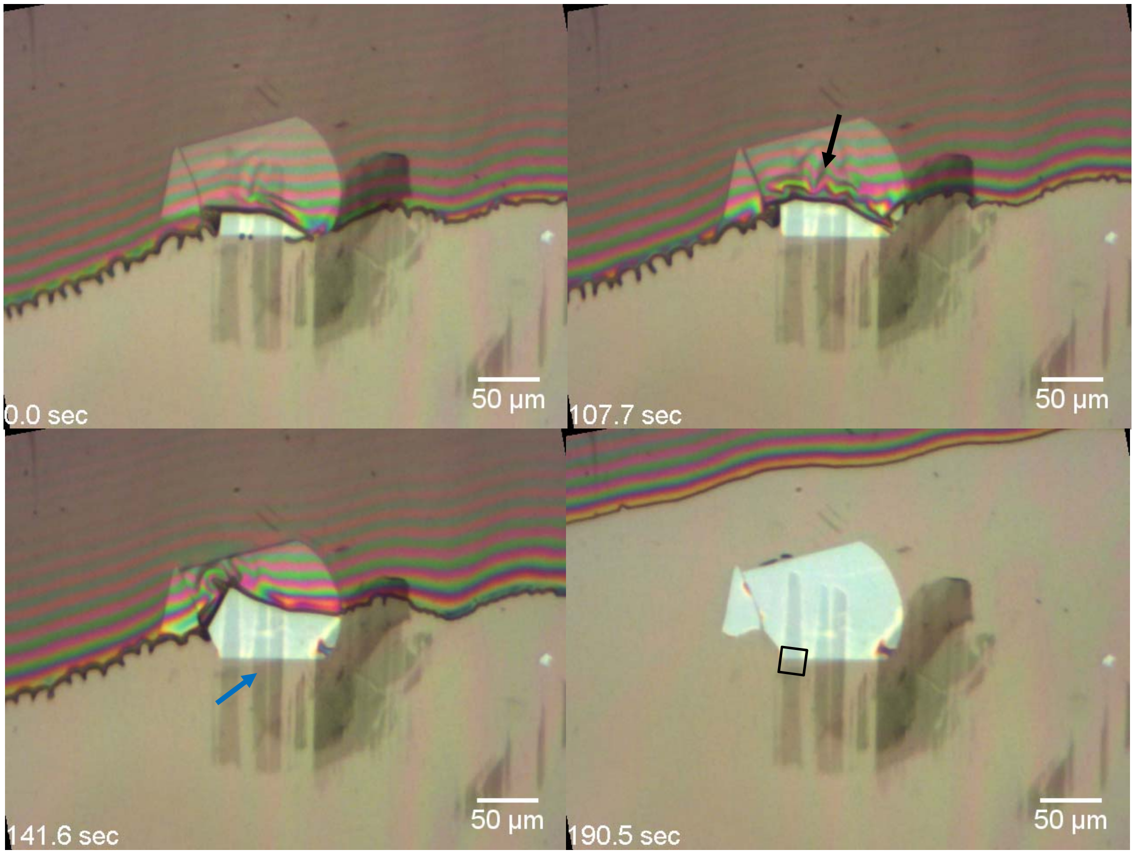

Supplementary Figure 14: Blister formation during drop-down. Drop-down of an hBN flake adhered to PPC/PDMS onto a graphene layer on SiO_2. In this instance, the contact front is not well controlled (black arrow), resulting in the visible formation of blisters (blue arrow) and inhomogeneous Raman *I(2D)/I(G)* and *G* peak FWHM values (See Supplementary Figure 6). The boxed region indicates where the data in Supplementary Figure 6 was acquired. Selected frames from Supplementary Movie 5)

Supplementary Methods

Details of the hot pick-up transfer are described in the following. Step 1: Pick up of the hBN flake from the silicon oxide surface. A piece of Si/SiO_2 with exfoliated hBN is placed on a microscope vacuum hot plate stage. The glass slide is held in a micromanipulator and kept approximately horizontal above the sample, with the polymer facing down. The PDMS/PPC is placed in contact with the hBN flake by lowering the glass slide along the *z*-axis of the micromanipulator. When the polymer completely covers the hBN flake, the temperature is raised to 55°C, and then lowered to 40°C. At this temperature, retracting the glass slide results in the pick-up of the hBN from the SiO_2 on the PPC (Figure 1 f i). This procedure is repeated for all the targeted hBN flakes (one per slide), so that a batch of hBN flakes adhered to glass slides is prepared.

Step 2: Drop-down of hBN on top of graphene. A Si/SiO_2 substrate with exfoliated graphene flakes is placed on the heating stage. The temperature is raised to 110 °C. A previously picked-up hBN flake is aligned over a chosen graphene flake with some separation between the surfaces (Figure 1 f ii). The glass slide is then lowered until the polymer comes into contact with the oxide surface. By further lowering the glass slide, the PPC front proceeds until reaching full contact with the SiO_2 surface. In this phase the polymer behaves as a liquid. It is important to proceed as slow as possible, in order to avoid non-conformal contact between hBN and graphene. When fully in contact, by moving slowly the glass slide away from the surface, the polymer front recedes very slowly. In this way it is possible to release the hBN from the PPC onto the graphene flake, without detaching the PPC from the PDMS block (Figure 1 f iii). The procedure is repeated until all the graphene flakes are covered by the hBN flakes prepared in 'Step 1'.

Step 3: baking. In order to promote the adhesion between graphene and hBN, the Si/SiO_2 substrate with the stacks is baked on a hot plate (in air) at 130 °C for 15 min.

Step 4: pick-up of the hBN/G stacks. All the hBN/G stacks are picked up from the SiO_2 surface repeating the procedure introduced in 'Step 1', one per glass slide (Figure 1 f iv).

Step 5: drop down of the hBN/G stack on the bottom hBN. Finally, the hBN/G stacks (on PPC) are dropped down on hBN flakes (cleaved on Si/SiO_2 substrates) similarly to what is done in 'Step 2' (Figure 1 f v-vii). The bottom hBN flakes are selected using dark field optical microscopy, as tape residues can be easily spotted and avoided.

Resistivity values for van der Pauw devices are measured by applying a constant source-drain bias to two neighbouring corner contacts of the device and measuring the voltage drop between the opposite contacts during sweeping of the gate voltage. This permits two pairs (through exchanging the source-drain and measurement terminals) of independent measurements of the resistance of the device $R^{1,2}_{\text{vertical}}$ and $R^{1,2}_{\text{horizontal}}$ across the orthogonal 'vertical' and 'horizontal' directions (Supplementary Figure 7), and the pairs are then averaged to produce R_{vertical} and $R_{\text{horizontal}}$. The sheet resistance R_S is calculated by applying the reciprocal van der Pauw formula $\exp(-\pi R_{\text{vertical}}/R_S) + \exp(-\pi R_{\text{horizontal}}/R_S) = 1$.
